# Time-Restricted Feeding and Intermittent Fasting as Preventive Therapeutics: A Systematic Review of the Literature

**DOI:** 10.7759/cureus.42300

**Published:** 2023-07-22

**Authors:** Arturo P Jaramillo, Javier Castells, Sabina Ibrahimli, Luisa Jaramillo, Rebeca R Briones Andriuoli, Denisse Moncada, Jhoanny C Revilla

**Affiliations:** 1 Internal Medicine, California Institute of Behavioral Neurosciences & Psychology, Fairfield, USA; 2 Internal Medicine, Universidad Católica de Santiago de Guayaquil, Guayaquil, ECU; 3 Cardiology, First Moscow State Medical University, Moscow, RUS; 4 Pediatrics, Maimonides Medical Center, New York, USA; 5 Internal Medicine, Universidad del Zulia, Maracaibo, VEN

**Keywords:** metabolically-unhealthy obese, ramadan diet plan for weight loss, caloric restriction, intermittent fasting, time restriction

## Abstract

Multiple studies have shown that intermittent fasting (IF) is associated with better health conditions and longer lifespans, as is time-restricted feeding (TRF). One crucial explanation is that IF and TRF permit a set length of time for caloric ingestion, during which our systems activate a variety of mechanisms that lead to the enhancement and renewal of different body systems. Accordingly, the benefits of IF and TRF are a lot greater than those of complete calorie restriction (CR). Accordingly, TRF and IF offered the underpinnings for human studies that revealed that when we eat and when we are fasting, we experience fluctuations in all body systems. For relevant medical literature, we investigated medical databases such as PubMed/Medline, PubMed Central, Cochrane Library, and Google Scholar. The chosen articles were evaluated based on eligibility criteria and vetted by quality evaluation methods; 15 finished research papers were included in the study. Of the 15 recognized studies, four were systematic reviews of literature, and 11 were review articles. The chosen publications all examined the efficacy and comparability with other restrictive diets. The study articles indicated that the advantages of IF and TRF represent complex interplay involving periodic digestion of food, gut flora, and the circadian clock. Accordingly, further research is necessary to get a comprehensive grasp of this very complex molecular blueprint. This could aid in producing an effectively planned food treatment that can regulate numerous chronic health ailments and disorders. Furthermore, it might lead to the development and investigation of new pharmacological medicines that mimic the nutritional and therapeutic benefits of IF for those who are unwilling or unable to follow this kind of feeding regimen.

## Introduction and background

Diverse intermittent fasting (IF) patterns have been studied in human and animal studies. This showed benefits for a variety of conditions, including glucose tolerance, obesity, cardiac conditions, liver alterations, dyslipidemia, and neurologic diseases [1]. All types of IF seem to be potential feeding choices for the treatment of numerous disorders impacting health and well-being. One of the explanations for IF's compatibility with human food patterns may be derived from historical studies of human dietary behaviors. Humans, like other animals, originated in habitats where food was limited, and as a result, they established feeding-fasting cycles dependent on food availability [1]. From a molecular perspective, IF may exert its benefits through a variety of mechanisms, such as reducing oxidative stress, optimizing circadian rhythms, and ketogenesis [2]. Meta-analytic studies may also have positive effects on body mass and adipose mass reduction [3].

In the United States alone, it is anticipated that the medical costs associated with overweight and obesity will exceed $90 billion. Reducing daily caloric consumption is the most frequently recommended method for promoting weight loss; current recommendations prescribe a combination of continuous energy restriction and a comprehensive change in lifestyle as the basis of obesity treatment [4]. Whereas cardiac mortality rates have improved, the drop in deaths has lately ended, and mortality among 35- to 64-year-old men and women in the United States has increased. Obesity and poor nutrition are significant modifiable factors contributing to the increase in cardiovascular disease, with an estimated 13% attributable risk of cardiovascular death [1-3]. Intermittent fasting is a dietary strategy that is comparable to calorie restriction (CR) in that it restricts food consumption. Intermittent fasting, on the other hand, focuses on the time when one may take meals within a day or a week. Alternate-day fasting and time-restricted fasting are the two broad categories of intermittent fasting. In alternate-day fasting, a subset may consist of 24-hour fasts followed by a 24-hour eating period that may be repeated multiple times per week, such as a 5:2 approach with two fast days and five nonrestrictive days. Variations for time-limited fast programs include 16-hour fasts with eight-hour meal schedules, 20-hour fasts with four-hour eating times, and other similar forms. While both calorie restriction and intermittent fasting may result in an overall lower caloric intake, intermittent fasting does not need this. In both people and animals, intermittent fasting has been associated with improved glucose management. Long-term adherence to calorie restriction, on the other hand, is poor, although intermittent fasting may be more promising [1,2]. Regardless of diet and macronutrient composition, CR adherence typically declines between one and four months [5].

An increase in the incidence of obesity, type II diabetes mellitus, and metabolic syndrome is linked to the excessive consumption of unhealthy foods such as high-fat, high-carbohydrate foods, and processed foods. IF increases lifespan and reduces the incidence of diseases associated with aging; some examples include overweight people, cardiovascular disease, cancer, renal conditions, and diabetes [1,3,6]. Aerobic exercise in conjunction with IF improved blood pressure lowering in individuals with limited medication response and has the potential to be introduced into regular therapy for these patients [4,6]. A randomized clinical study found that, compared to usual treatment, 12 weeks of moderate-intensity aerobic exercise training plus intermittent fasting (IF) lowered 24-hour systolic blood pressure by 7.1 mm Hg in people with resistant hypertension. Daytime blood pressure (systolic, 8.4 mm Hg; diastolic, 5.7 mm Hg), 24-hour diastolic blood pressure (5.1 mm Hg), and office systolic blood pressure (10.0 mm Hg) were all considerably lower following exercise training compared to standard care [2,3,6]. IF is associated with significant body fat reduction that leads to weight loss over approximately eight to twelve weeks, along with the management of lipid problems, alterations in body composition, and hypertension [6].

## Review

Methodology

We did a systematic evaluation using the Preferred Reporting Items for Systematic Reviews and Meta-Analyses (PRISMA) 2020 guidelines to describe our approach and results. 

Study Duration

This review started on June 10th, 2023.

Search Strategy

PubMed, Google Scholar, and Cochrane library were used to collect database using the following: (("Intermittent Fasting/blood"(Majr) OR "Intermittent Fasting/metabolism"(Majr) OR "Intermittent Fasting/physiology"(Majr))) OR ("Intermittent Fasting/blood"(Majr:NoExp) OR "Intermittent Fasting/metabolism"(Majr:NoExp) OR "Intermittent Fasting/physiology"(Majr:NoExp)) AND (("Caloric Restriction/adverse effects"(Majr) OR "Caloric Restriction/methods"(Majr) OR "Caloric Restriction/trends"(Majr))) OR ("Caloric Restriction/adverse effects"(Majr:NoExp) OR "Caloric Restriction/methods"(Majr:NoExp) OR "Caloric Restriction/trends"(Majr:NoExp)) AND (("Fasting/adverse effects"(Majr) OR "Fasting/metabolism"(Majr) OR "Fasting/physiology"(Majr))) AND ("Fasting/adverse effects"(Majr:NoExp) OR "Fasting/metabolism"(Majr:NoExp) OR "Fasting/physiology"(Majr:NoExp)).

Eligibility Criteria and Study Selection

To assess eligibility, two investigators carefully read the full title and content of each paper. We selected the latest literature and articles published in the past five years, including papers written in the English language or if a full free-text English-language translation is available. Articles were excluded if the full free text of the papers could not be retrieved. Articles focusing on the different diets used as optional therapeutics for inflammatory bowel disease (IBD) gray literature and proposal papers were also not included.

Data Management

Two independent writers evaluated papers based on titles and abstracts. Following that, significant abstracts were examined for a complete, free full-text examination. The selected studies were evaluated, and if there was any dispute, the research was evaluated by a third author. Information from the relevant publications was then collected. The first author's name, type, year of publication, study design, and results were taken as priorities. Finally, duplicates were deleted.

Quality Assessment

We used the assessment of multiple systematic reviews (AMSTAR) quality tool for systematic review literature and the scale for the assessment of narrative review articles (SANRA) quality tool for literature review (LR).

Results

Search Results

**Figure 1:**
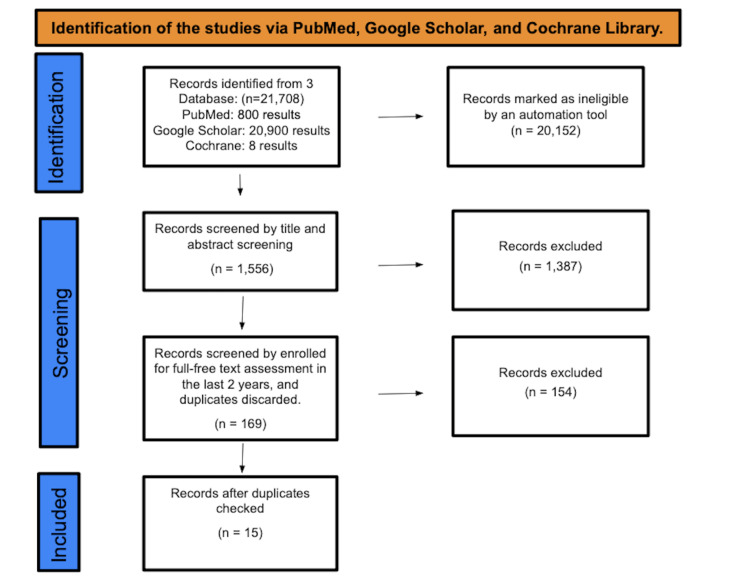
Identification of studies via databases and registers.

A total of 21,708 studies were found after searching the aforementioned databases; 20,152 were marked as ineligible by an automation tool. There were a total of 1,556 studies that underwent a selection by title and abstract screening, with 1,387 papers being discarded. The remaining 169 papers were chosen by full-free text evaluation in the previous two years, and after discarding duplicates, resulting in the elimination of 154 studies, only 15 studies were enlisted for the final collection of data (Figure 1).

Table 1 summarizes and characterizes all investigations.

**Table 1:** Table of data extraction. IER: intermittent energy restriction; LR: literature review; RCT: randomized clinical trials; SRL: systematic literature review; CT: clinical trials; IF: intermittent fasting; CR: caloric restriction; ADF: alternate-day fasting; T2DM: type 2 diabetes mellitus; TRF: time-restricted feeding, LDL: low-density lipoprotein; SANRA: scale for the assessment of narrative review articles; AMSTAR: assessment of multiple systematic reviews.

Author	Year of publication	Study design	Quality tool	Primary research	Outcome evaluation
Song et al. [7]	2022	LR	SANRA checklist	Various articles were gathered and reviewed; among them were RCTs, SRLs, and meta-analyses.	In terms of decreasing insulin effects, IF outperformed CR. A thorough knowledge of the processes behind the therapeutic benefits of IF opens up opportunities for customized dietary regimens to address a broad variety of illnesses and health problems.
Zhang et al. [8]	2022	SRL and meta-analysis	SANRA checklist	SRL and meta-analyses of randomized clinical trials and pilot trial studies were used to evaluate the efficacy of intermittent fasting versus continuous calorie restriction in overweight and obese adults.	IF as a benefit for weight loss was significant, especially in patients with sedentary lives that for them are hard to attach to an exercise lifestyle.
Vasim et al. [9]	2022	LR	SANRA checklist	Articles about how fasting at different times affects the body, especially in the regimen of IF time (5:2, 16:8, 20:4)	Several small-scale investigations on humans have demonstrated the significance of a time-restricted dietary pattern for the maintenance of a healthy metabolism.
Altay [10]	2022	LR	SANRA checklist	The evidence for IF uses in diabetes will be thoroughly explored and reviewed from different studies, including RCTs, SRLs, and meta-analyses.	The benefits of IF outweigh the risks for diabetic individuals; this was seen more in patients with no compliance with their medication as well as those with low capability for a more active lifestyle.
Varady et al. [11]	2021	LR	SANRA checklist	An examination of the impact of several fasting regimens, including ADF, the 5:2 diet, and TRF, on various health markers in human volunteers.	In conclusion, IF is a safe diet treatment that may help obese individuals reduce clinically significant amounts of weight (>5%) and improve several signs of metabolic health.
Morales-Suarez-Varela et al. [12]	2021	SRL	AMSTAR checklist	Fifteen RCTs and 16 LRs were selected as per the subject of interest in the IF and CR diets and their benefits.	Both IF and CR diets have almost the same outcomes in T2DM and obesity, especially in lipid profile outcomes, Hb1Ac, and reductions in inflammatory markers.
Duregon et al. [13]	2021	LR	SANRA checklist	Current status of knowledge on different strategies to reap the benefits of CR on metabolic health in humans	In both short- and long-term human trials, IF has been associated with metabolic alterations, including reduced body weight and adipose mass, decreased blood glucose, and enhanced insulin sensitivity.
Welton et al. [14]	2021	SRL	AMSTAR checklist	Weight loss in overweight and obese people was addressed in 41 papers summarizing 27 studies: 18 small RCTs and nine trials comparing weight after IF to baseline weight with no control group.	IF demonstrates promise as an obesity treatment. In all 27 trials examined, IF resulted in weight loss ranging from 0.8% to 13.0% of baseline body weight.
Ismail et al. [15]	2020	LR	SANRA checklist	There are almost 40 published articles from 1970 to 2019. The publications were found using several databases, including PubMed/Medline, Science Direct, PLoS One, Scopus, the Directory of Open Access Journals, and the Cochrane Library.	Fasting during Ramadan looks to have a major influence on weight loss and LDL cholesterol, which might lead to considerable reductions in cardiovascular illnesses, such as coronary heart disease, myocardial diseases, and atherosclerosis.
Hu et al. [1]	2020	LR	SANRA checklist	Medline, Embase, Cochrane Library, and Google Scholar were used to search for IF and TRF-related studies.	The advantage of IF over CR is that IF promotes the diversity of our intestinal flora, which restores a circadian rhythm comparable to that of our ancestors.
Dong et al. [2]	2020	LR	SANRA checklist	The present literature on the possible cardiovascular advantages of IF is reviewed, and future study options are suggested.	In human studies, IF seems to have cardiovascular advantages. IF has been linked to a better prognosis after a cardiac attack.
Correia et al. [3]	2020	SRL	AMSTAR checklist	Specific outcomes of IF on exercise performance. Twenty-eight articles met the eligibility criteria.	IF may also have some beneficial effects on body mass and fat mass. The pooled estimates of the effects of IF on muscular strength and anaerobic ability were mostly non-significant.
Rynders et al. [4]	2019	LR	SANRA checklist	Evidence supports intermittent energy restriction (IER) regimens as therapies for obesity and overweight. RCT of eight weeks length in people with overweight or obesity (BMI 25 kg/m^2^) in which an IER paradigm (IF or TRF) was compared against CR, with weight reduction as the main endpoint.	The nutrition community should aim toward standardized approaches for monitoring meal intake timing in IER and specifically TRF research.
Lopes et al. [6]	2019	SRL	AMSTAR checklist	The research included 53 patients, including 26 in the exercise group and 27 in the control group. There were 24 women among them, with a mean age of 60.	In individuals with resistant hypertension, a 12-week aerobic exercise program in combination with IF lowered 24-hour and daytime ambulatory blood pressure as well as office systolic blood pressure.

Discussion

According to Jebeile et al.’s LR, numerous studies have shown that IF is a beneficial and manageable strategy for obese individuals, particularly obese adolescents [16]. CR has been a key treatment modality for obesity for a long time; recently, IF has been considered an alternative dietary approach to CR; people who practice IF say that doing this is easier than CR [7].

Santos et al.’s thorough review and meta-analysis indicated that IF is a helpful lifestyle change because it enhances the lipid profile and reduces body weight [17]. According to research, eight consecutive weeks of ADF led to a 6.8% decrease in blood glucose levels after fasting and a 22% decrease in insulin concentrations in obese individuals [11]. Due to the depletion of liver glycogen stores during fasting, ADF increases blood lipid levels while decreasing body mass and body weight; triglyceride levels suggest that free fatty acids circulate into liver cells to produce ketone energy [18].

In 2017, Trepanowski et al. compared ADF versus daily CR on weight loss, weight maintenance, and cardiovascular disease risk indicators, which demonstrate the high benefits of ADF versus CR [19]. Duregon et al. found that the beneficial effects of IF on obesity are at least in part due to the shift from glucose to fatty acids and ketones as the preferred fuel source [13]. Trepanowski et al. mentioned that in other studies after one year, the scientists found no health advantages from ADF beyond calorie reduction [19].

Antay et al.'s LR study demonstrated how Arnason et al. implemented meal restriction for about 20 hours for fourteen days in ten metformin-treated type 2 diabetes obese patients. As a result, patients' blood glucose and weight decreased after the IF regimen, but there was no discernible change in the homeostatic model assessment of insulin resistance (HOMA-IR) (a measure of insulin resistance and techniques to reduce it) or serum lipid levels [20]. In a 52-week randomized controlled trial, Carter et al. randomly assigned 137 obese type 2 DM patients to either a continuous energy restriction group or a 5:2 IER group. After the study, both groups demonstrated a significant decrease in Hba1c, fasting glucose, body weight, and LDL levels. Additionally, a significant reduction in insulin levels was noticed in both groups [21].

Harvie et al.'s LR revealed that both diastolic and systolic blood pressure decreased after twenty-four to forty weeks of intervention with a 5:2 IF, and the reductions were comparable to those of CR [22]. In two studies, LDL cholesterol and triglyceride rates improved over time [21]. According to a meta-analysis conducted by Morales-Suarez-Varela et al., IF and short-term CR diets had almost the same outcome in weight loss in adults with obesity and individuals with type 2 diabetes [12]. There are few long-term clinical studies, but these have demonstrated that IF is preferable to CR in reducing waist measures and central fat distribution, but both are still important for reducing cardiovascular risk [21]. An LR conducted by de Cabo et al. demonstrated that prolonging the time expended in the fasting state enhances metabolic homeostasis and protects against cardiovascular, neoplastic, and neurodegenerative conditions in humans [23]. A three-month study comparing daily CR versus IF (5:2 dietary regimen) in obese women aged 20 to 69 revealed that while both resulted in a significant reduction in body weight, the 5:2 diet resulted in a greater reduction in body fat percentages and a boost in insulin sensitivity [24].

In a randomized controlled trial, Welton et al. found that IF is a moderately effective method for weight loss and has the potential to improve glycemic control. The majority of participants in these studies had type 2 diabetes [20,25]. In initial research conducted in 2016, Carter et al. compared two fasting days a week with an otherwise normal diet to daily calorie restriction, showing a reduction in body weight and body fat percentage in the two fasting day groups in comparison with the control group [25]. A 2013 review of the literature by Gasmi et al. found that fasting may be an option for reducing systemic low-grade inflammation and age-related degenerative diseases associated with immune senescence without impairing physical performance [26].

## Conclusions

We may infer that IF and TRF can be utilized therapeutically and preventively after examining and choosing the most relevant information. However, because of the limited amount of research, it has been overestimated. IF and TRF may be utilized specifically in patients with T2DM and obesity; however, it is believed because of the idiosyncrasy of the mind when the phrase "do not eat for a certain time" is used, leading to the belief that it means "not having good health". Several of the investigated studies provide us with alternative viewpoints on how to target IF and TRF not only to reduce weight but also for a variety of other ailments such as cancer, immunological and gastroenterological diseases, and aging, among others. Even reading an article on the renowned Ramadan diet led to the conclusion that a short period of IF resulted in a change in the quality of life. However, IF and TRF have not been extensively researched in long-term trials to provide additional evidence for their dubious advantages. It highlights the need for further long-term research to establish the specificities of IF and TRF that may be used as preventative therapy regimens for a variety of illnesses in the future.

## References

[1] Hu D, Xie Z, Ye Y, Bahijri S, Chen M (2020). The beneficial effects of intermittent fasting: an update on mechanism, and the role of circadian rhythm and gut microbiota. Hepatobiliary Surg Nutr.

[2] Dong TA, Sandesara PB, Dhindsa DS (2020). Intermittent fasting: a heart healthy dietary pattern?. Am J Med.

[3] Correia JM, Santos I, Pezarat-Correia P, Minderico C, Mendonca GV (2020). Effects of intermittent fasting on specific exercise performance outcomes: a systematic review including meta-analysis. Nutrients.

[4] Rynders CA, Thomas EA, Zaman A, Pan Z, Catenacci VA, Melanson EL (2019). Effectiveness of intermittent fasting and time-restricted feeding compared to continuous energy restriction for weight loss. Nutrients.

[5] Dansinger ML, Gleason JA, Griffith JL, Selker HP, Schaefer EJ (2005). Comparison of the Atkins, Ornish, Weight Watchers, and Zone diets for weight loss and heart disease risk reduction: a randomized trial. JAMA.

[6] Lopes S, Mesquita-Bastos J, Garcia C (2021). Effect of exercise training on ambulatory blood pressure among patients with resistant hypertension: a randomized clinical trial. JAMA Cardiol.

[7] Song DK, Kim YW (2023). Beneficial effects of intermittent fasting: a narrative review. J Yeungnam Med Sci.

[8] Zhang Q, Zhang C, Wang H (2022). Intermittent fasting versus continuous calorie restriction: which is better for weight loss?. Nutrients.

[9] Vasim I, Majeed CN, DeBoer MD (2022). Intermittent fasting and metabolic health. Nutrients.

[10] Altay M (2022). Evidence-based information about intermittent fasting in diabetes patients: useful or harmful?. Turk J Med Sci.

[11] Varady KA, Cienfuegos S, Ezpeleta M, Gabel K (2021). Cardiometabolic benefits of intermittent fasting. Annu Rev Nutr.

[12] Morales-Suarez-Varela M, Collado Sánchez E, Peraita-Costa I, Llopis-Morales A, Soriano JM (2021). Intermittent fasting and the possible benefits in obesity, diabetes, and multiple sclerosis: a systematic review of randomized clinical trials. Nutrients.

[13] Duregon E, Pomatto-Watson LC, Bernier M, Price NL, de Cabo R (2021). Intermittent fasting: from calories to time restriction. Geroscience.

[14] Welton S, Minty R, O’Driscoll T (2020). Intermittent fasting and weight loss: systematic review. Can Fam Phys.

[15] Ismail A, Mohamoud A, Mussa M (2020). Ramadan intermittent fasting and its beneficial effects of health: a review article. Cent Afr J Public Health.

[16] Jebeile H, Gow ML, Lister NB (2019). Intermittent energy restriction is a feasible, effective, and acceptable intervention to treat adolescents with obesity. J Nutr.

[17] Santos HO, Macedo RC (2018). Impact of intermittent fasting on the lipid profile: assessment associated with diet and weight loss. Clin Nutr ESPEN.

[18] Ng GY, Kang SW, Kim J (2019). Genome-wide transcriptome analysis reveals intermittent fasting-induced metabolic rewiring in the liver. Dose Response.

[19] Trepanowski JF, Kroeger CM, Barnosky A (2017). Effect of alternate-day fasting on weight loss, weight maintenance, and cardioprotection among metabolically healthy obese adults: a randomized clinical trial. JAMA Intern Med.

[20] Arnason TG, Bowen MW, Mansell KD (2017). Effects of intermittent fasting on health markers in those with type 2 diabetes: a pilot study. World J Diabetes.

[21] Carter S, Clifton PM, Keogh JB (2018). Effect of intermittent compared with continuous energy restricted diet on glycemic control in patients with type 2 diabetes: a randomized noninferiority trial. JAMA Netw Open.

[22] Harvie MN, Pegington M, Mattson MP (2011). The effects of intermittent or continuous energy restriction on weight loss and metabolic disease risk markers: a randomized trial in young overweight women. Int J Obes (Lond).

[23] de Cabo R, Mattson MP (2019). Effects of intermittent fasting on health, aging, and disease. N Engl J Med.

[24] Harvie M, Wright C, Pegington M (2013). The effect of intermittent energy and carbohydrate restriction v. daily energy restriction on weight loss and metabolic disease risk markers in overweight women. Br J Nutr.

[25] Carter S, Clifton PM, Keogh JB (2016). The effects of intermittent compared to continuous energy restriction on glycaemic control in type 2 diabetes; a pragmatic pilot trial. Diabetes Res Clin Pract.

[26] Gasmi M, Sellami M, Denham J, Padulo J, Kuvacic G, Selmi W, Khalifa R (2018). Time-restricted feeding influences immune responses without compromising muscle performance in older men. Nutrition.

